# Mid- to Long-Term Clinical Outcomes After Lumbar Microdiscectomy in Adolescent Patients: A Descriptive Study

**DOI:** 10.3390/children13040578

**Published:** 2026-04-21

**Authors:** Lawrence Sanchez, Johanna Austeen Gjestland, Per-Henrik Randsborg, Ole Kristian Alhaug

**Affiliations:** 1 Department of Orthopedic Surgery, Ålesund Hospital, 6017 Ålesund, Norway; 2 Department of Orthopedic Surgery, Akershus University Hospital, 1478 Lørenskog, Norway; johanna.austeen.gjestland@ahus.no (J.A.G.); per-henrik.randsborg@ahus.no (P.-H.R.); olekralhaug@hotmail.com (O.K.A.); 3 Institute of Clinical Medicine, Faculty of Medicine, University of Oslo, 0316 Oslo, Norway

**Keywords:** adolescent, lumbar disc herniation, microdiscectomy, Oswestry Disability Index

## Abstract

**Highlights:**

**What are the main findings?**

- Lumbar microdiscectomy showed acceptable mid- to long-term outcomes in adolescents.
- Mean ODI was 9.5 at more than five years of follow-up.

**Abstract:**

**Background/Objectives**: Adolescent lumbar disc herniation (ALDH) is rare. Evidence on long-term surgical outcomes remains limited. The objective of this study was to evaluate mid- and long-term clinical outcomes following lumbar microdiscectomy in adolescents with lumbar disc herniation (LDH). **Methods**: A single-institution retrospective cohort study of patients under 19 years who underwent lumbar microdiscectomy over a ten-year period. Baseline clinical and radiological data were obtained from electronic patient records (EPR) and Magnetic Resonance Imaging (MRI). Patient-reported outcomes were collected at follow-up using the Oswestry Disability Index (ODI) as the primary outcome and Numeric Rating Scales (NRS) for back and leg pain and Global Perceived Effect (GPE) as secondary outcomes. Descriptive statistics were used to summarize results. **Results**: Seventeen of 27 patients (63%) participated. Mean age at surgery was 16.9 years, and mean symptom duration prior to surgery was 11.3 months. All patients underwent disc-preserving microdiscectomy. At a mean follow-up of 67.7 months, mean ODI was 9.5, mean NRS back pain was 2.8, and mean NRS leg pain was 2.3. Fourteen patients (82%) reported being completely recovered or much improved. **Conclusions**: Lumbar microdiscectomy in adolescents with LDH showed acceptable mid- to long-term outcomes, low disability, and low pain levels at more than five years of follow-up. Clinical and imaging findings resembled those seen in adults, though symptom duration before surgery was prolonged.

## 1. Introduction

Lumbar disc herniation (LDH) is among the most common indications for spinal surgery in adults, with peak incidence in the fourth and fifth decades of life [1,2,3]. In contrast, LDH is relatively uncommon in children and adolescents, and its true prevalence remains poorly defined.

Adolescent lumbar disc herniation (ALDH) typically presents with clinical features similar to those observed in adults. However, the underlying etiology often differs. Whereas LDH in adults is primarily associated with degenerative disc disease, ALDH is more frequently linked to trauma, high-impact sports, or congenital spine anomalies. Diagnosing ALDH can be challenging due to its low incidence, variability in clinical presentation, and the relative reluctance to perform advanced imaging in pediatric populations. Management strategies range from conservative management to surgical intervention, with microdiscectomy being the most commonly reported surgical procedure. However, the long-term outcomes of surgical treatment for LDH in adolescents remain insufficiently characterized. Most available studies are limited by small sample sizes, variable follow-up duration, and heterogeneous study designs [4].

This study aimed to report the mid- and long-term clinical outcomes of lumbar microdiscectomy in adolescents.

## 2. Materials and Methods

This single-institution retrospective cohort study included adolescents who underwent lumbar microdiscectomy at Akershus University Hospital (Ahus) between January 2014 and April 2024. Ahus is the largest emergency hospital in Norway, serving a population of approximately 627,000 inhabitants. The spine surgery unit consists of seven consultant orthopedic spine surgeons and performs approximately 530 surgeries annually. Study participants were identified through the hospital database by selecting patients under 19 years of age diagnosed with LDH and radiculopathy (International Classification of Diseases, Tenth Revision (ICD-10) code M51.1) who underwent lumbar microdiscectomy of lumbar intervertebral disc displacement (NOMESCO Classification of Surgical Procedures (NCSP) code ABC16).

### 2.1. Data Collection

**Figure 1:**
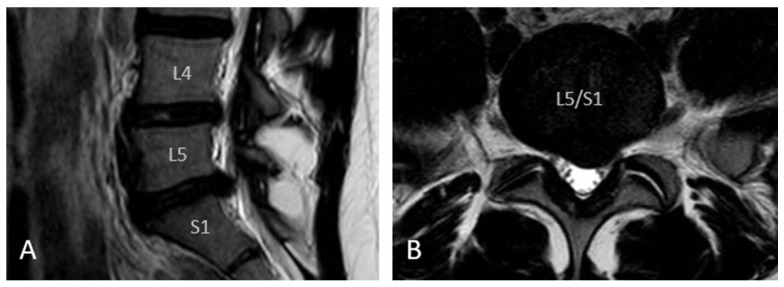
Sagittal (**A**) and axial (**B**) T2-weighted MRI of an adolescent patient showing a left broad-based L5/S1 disc herniation. Informed consent was obtained from the patient for publication of this image.

The electronic patient records (EPR) were reviewed for age, gender, medical history, etiology of LDH, clinical findings, duration of symptoms, radiological findings, level of activity, and reoperations. The Magnetic Resonance Imaging (MRI) images (an example shown in Figure 1) were examined by the first author for the level of disc herniation, disc herniation morphology, and the presence of disc degeneration. The Modified Pfirrmann grading system was used to assess disc degeneration [5].

### 2.2. Surgical Procedure

Indications for surgery were failed conservative treatment of more than 3 months, with persistent leg pain correlating with lumbar disc herniation on MRI. The decision to operate was reached in a shared decision process with the patients and their legal guardians.

Lumbar microdiscectomy was performed through a small posterior incision under the microscope. The paraspinal muscles were retracted with Caspar self-retaining retractors to expose the laminae. A limited laminotomy and removal of the ligamentum flavum were performed to identify the compressed nerve root, which was gently mobilized to expose and remove the herniated disc fragment. The procedure is considered an open, disc-preserving approach, as subperiosteal muscle detachment is used and no aggressive or subtotal discectomy is performed. Finally, the wound was irrigated and closed.

Postoperative rehabilitation was unrestricted. No systematic physiotherapy or formal clinical follow-up was performed.

### 2.3. Outcomes

Patients were invited electronically and provided informed consent via a secure online form before completing the questionnaire.

The primary outcome measure was the Oswestry Disability Index (ODI) (Norwegian translation), version 2.0. ODI is one of the most widely used patient-reported outcome measures (PROM) for assessing disability related to back pain. It includes ten questions covering activities of daily living, each with five response options scored 0–5. The responses are summed and converted into a percentage score ranging from 0% (minimal disability) to 100% (completely disabled/bed-bound) [6,7].

The secondary outcomes included Numeric Rating Scales (NRS) for back and leg pain and Global Perceived Effect (GPE) to measure overall clinical change. The NRS ranges from 0 (no pain) to 10 (worst imaginable pain). The NRS is simple to use, correlates well with other pain-measuring tools, and is recommended for measuring chronic pain [8,9]. GPE is a seven-point Likert scale (1 = “Completely recovered”, 2 = “Much improved”, 3 = “Somewhat improved”, 4 = “Unchanged”, 5 = “Somewhat worse”, 6 = “Much worse”, 7 = “Worse than ever”) [10].

Additional questions were included regarding the level and type of physical activity before the onset of disc herniation, the level of physical activity at follow-up, the need for further spinal surgery beyond the index surgery, and school absence at follow-up.

### 2.4. Statistics

Descriptive statistics were used to summarize the data. Continuous data are presented as means and 95% confidence intervals (CI), and categorical data are presented as numbers and proportions (%). We used SPSS version 30 (IBM Corp., Armonk, NY, USA) and Excel (Microsoft 365 MSO, version 2510 Build 16.0.19328.20178).

### 2.5. Ethics

The project was approved by the Regional Committee for Medical and Health Research Ethics (REK) (application number 759291) and the Data Protection Officer at Ahus. Informed consent was obtained before the study began. All patients provided written consent to participate in the study.

## 3. Results

Of the 27 patients identified, 17 (63%) consented to participate and were included in the study. The mean age at the time of surgery was 16.9 years (95% Confidence Interval (CI): 16.3–17.5), with the youngest patient being 14 years old. Among the included patients, 15 (88%) presented with leg pain, and 11 (65%) also reported lower back pain. The mean duration of symptoms prior to surgery was 11.3 months (95% CI: 7.4–15.2).

Index-level disc degeneration was observed in all patients. Baseline characteristics are summarized in Table 1. All patients underwent disc-preserving microdiscectomy. No perioperative complications were reported.

At a mean follow-up of 67.7 months (95% CI: 48.6–86.8) after surgery, the mean ODI was 9.5 (95% CI: 4.58–14.36), mean NRS back pain was 2.81 (95% CI: 1.67–3.95), and mean leg pain was 2.31 (95% CI: 1.35–3.28). The mean age at follow-up was 21.8 years (95% CI: 19.9–23.7). Fourteen patients (82%) reported complete recovery or much improved. Seven patients (41%) were less active than before symptom onset, with five (29%) attributing this to the disc herniation. Within six months, two patients (12%) experienced recurrent lumbar disc herniation, and one (6%) underwent repeat surgery. The follow-up data are presented in Table 2.

Appendix A, Table A1 shows individual patient characteristics and postoperative outcome measures, while Table A2 presents activity level prior to disc herniation.

## 4. Discussion

In this mid- to long-term follow-up of 17 adolescents who underwent surgical treatment for LDH, most patients reported acceptable functional outcomes. The mean ODI was 9.5, and both back and leg pain scores were low. Fourteen patients (82%) reported being much improved or fully recovered. To our knowledge, this study is among the first to report long-term (greater than five years) pain outcomes in adolescents undergoing surgery for LDH.

Although the available literature on ALDH is limited, our findings are generally consistent with previous reports. Karademir et al. reported a 1-year mean ODI of 9.9, while Smorgick et al. observed excellent or good outcomes (ODI < 20) in 65% of patients at mid- to long-term follow-up [11,12]. The mean ODI of 9.5 observed in our cohort is lower (indicating better function) than values reported for the general adult population (14.4) and for adults with degenerative spine conditions or LDH (approximately 17–20) [13,14]. Similarly, the mean NRS scores for back and leg pain (2.81 and 2.31, respectively) were comparable to those reported in adult surgical populations [15]. The proportion of patients reporting clinical improvement (82%), corresponding to responses of ‘completely recovered’ or ‘much improved’ on the GPE scale, was also similar to outcomes described in adult cohorts undergoing surgery for LDH [14]. However, these comparisons should be interpreted with caution due to differences in study design, populations, and follow-up durations.

We observed a mean of 11 months from symptom onset to surgery and 4 months from symptom onset to MRI. The 11-month interval lies at the upper end of previously reported ranges (7–12 months) [11,16,17]. This prolonged interval likely reflects the Norwegian care pathway—initial general practitioner evaluation with referral to imaging and specialists only after persistent symptoms—as well as caution regarding surgery in young patients and local capacity constraints.

While an 11-month duration represents a substantial period for adolescents, particularly those experiencing limitations in school attendance or sports participation, this study was not designed to evaluate the impact of the timing of diagnosis or intervention on outcomes. Further investigation is required to clarify whether symptom duration influences long-term results in this population.

Trauma has frequently been cited as a risk factor for adolescent lumbar disc herniation, with reported rates ranging from 30% to 60% [4,18,19]. In contrast, only 12% of patients in our cohort reported a history of trauma. This discrepancy may be due to underreporting or incomplete EPR documentation.

Most herniations in our cohort were located at the L5/S1 and L4/L5 levels, consistent with previous studies and comparable to patterns observed in adult populations [11,20]. All patients demonstrated index-level disc degeneration on imaging, a higher proportion than reported by Karademir et al., who observed degeneration in 63% of cases [11]. Additionally, nearly half of the patients (47%) showed MRI evidence of adjacent-level disc degeneration. To our knowledge, adjacent-level degeneration has not been widely reported in studies of ALDH, and the clinical significance of this finding remains uncertain.

The majority of patients reported both back and leg pain, mirroring the clinical presentation commonly seen in adults with LDH [17,18]. This suggests that the symptomatic presentation of ALDH may be similar to that observed in adult patients. The low frequency of reported trauma, the distribution of affected spinal levels, and the high frequency of disc degeneration observed in this cohort may support the hypothesis that early-onset disc degeneration contributes to the development of ALDH [21,22]. However, a direct etiological role cannot be established based on the present findings.

At follow-up, seven patients (41%) reported reduced activity levels, which contrasts with the generally acceptable ODI scores. This discrepancy may reflect several factors. First, ODI primarily captures disability related to daily functioning and may not be sensitive to changes in higher-level physical activity or sports participation. Second, interpretation of activity data is limited, as no validated instruments for assessing activity levels in this population were used. Third, cessation of organized physical activity is common during adolescence. A Norwegian population-based study has shown that ages 13 to 18 years represent a period when adolescents frequently discontinue organized activities, independent of medical conditions [23]. Finally, the prolonged duration of symptoms prior to surgery may have contributed to behavioral adaptations or deconditioning in some patients. Thus, reduced activity levels likely reflect a combination of residual symptoms, lifestyle changes, and age-related factors rather than persistent disability alone.

Whether earlier diagnosis or intervention influences outcomes in this population warrants further study.

### Limitations

This study has several limitations. First, the lack of a control group limits conclusions about the natural course of ALDH. Although our first line of treatment is non-operative for the majority of patients, we do not know how our patients would have fared with continuous non-operative management. Furthermore, the true effect of the surgical procedure may be overestimated, either due to regression to the mean or the placebo effect of the surgery. The absence of baseline PROMs, such as ODI and NRS, further restricts the ability to assess the true magnitude of improvement. This highlights the need for future trials with appropriate controls or registry-based comparative studies, including patients managed conservatively.

Secondly, the cohort was small, and 37% of eligible patients declined participation, though the sample size is comparable to previous studies on adolescent lumbar disc herniation [12,24]. The non-responders in our study did not differ from responders with respect to age but were predominantly female. Two patients among the non-responders underwent emergency surgery due to neurologic deficits. All patients underwent open lumbar microdiscectomy. Baseline data on non-responders are limited due to the lack of informed consent. Available baseline characteristics of non-responders are summarized in Appendix A, Table A3.

Third, it is possible that some patients were not identified during the hospital database search, thereby potentially excluding adolescents who underwent lumbar spinal surgery classified differently by NCSP.

Finally, a limitation of this study is the use of adult patient-reported outcome measures (ODI and NRS), which have not been formally validated in adolescents. This limitation is well recognized in the literature on pediatric PROMs, where measurement validity and interpretation of outcomes can be problematic, particularly for younger children [25,26]. However, our cohort consisted mainly of older adolescents, and all patients except three had reached adulthood at the time of follow-up. Nevertheless, as no PROMs specifically validated for adolescents with lumbar disc herniation are currently available, we included questions on school attendance and sports participation, which reflect issues more relevant to younger patients.

## 5. Conclusions

This study showed acceptable outcomes after surgical treatment for adolescent lumbar disc herniation, with a mean ODI score of 9.5 at a mean follow-up of 5.6 years. Clinical and imaging findings mirrored those in adults, but the time from symptom onset to surgery was prolonged.

## Figures and Tables

**Table 1:** Baseline data for 17 ALDH patients at Akershus University Hospital from January 2014 to April 2024.

		Mean/Number	95% CI/Proportion (%)
Age (years)		16.9	16.3–17.5
Females		8	47.1%
BMI kg/m^2^		24	22.3–25.7
Patients with comorbidities		4	23.5%
Known triggering cause		7	41.2%
	High-energy trauma	2	11.8%
Symptoms			
	Leg pain	15	88.2%
	Low back pain	11	64.7%
	Paresthesia	5	29.4%
	Paresis	0	-
Disc herniation segment			
	L5/S1	11	64.7%
	L4/L5	6	35.3%
Disc herniation type			
	Broad-based	14	82.4%
	Focal	2	11.8%
	Bulge	1	5.9%
	Foraminal	0	-
Disc degeneration			
	Index level	17	100%
	Adjacent level	8	47.1%
Duration of symptoms prior to surgery (months)		11.3	7.4–15.2
Time from MRI to surgery (months)		4.2	1.7–6.7

ALDH Adolescent lumbar disc herniation, CI Confidence Interval, BMI Body Mass Index, Comorbidities (thrombocytopenia, anxiety, depression, gastroesophageal reflux disease, eczema, hypothyreosis), MRI Magnetic Resonance Imaging.

**Table 2:** Follow-up (mean 67.7 months, 95% CI: 48.6–86.8) data after surgical treatment for 17 ALDH patients at Akershus University Hospital from January 2014 to April 2024.

		Mean/Number	95% CI/Proportion (%)
ODI		9.47	4.58–14.36
NRS leg pain		2.31	1.35–3.28
NRS back pain		2.81	1.67–3.95
GPE			
	1—Completely recovered	5	29.4%
	2—Much improved	9	52.9%
	3—Somewhat improved	1	5.9%
	4—Unchanged	2	11.8%
	5—Somewhat worse	0	-
	6—Much worse	0	-
	7—Worse than ever	0	-
Activity level at follow-up			
	Normal activity; no organized sports team	6	35.3%
	Exercise regularly 1–3 times per week; no organized sports team	5	29.4%
	Exercise regularly 1–3 times per week; affiliated with sports team	3	17.6%
	Exercise more than 3 times per week; affiliated with sports team	2	11.8%
School attendance at follow-up			
	Do not attend school due to pain	1	5.9%
	Absent 1–3 days per week due to pain	0	-
	Absent 1–3 days per month due to pain	0	-
	Absent 1–3 days per year due to pain	2	11.8%
	Not absent due to pain	14	82.3%
Additional surgery for LDH		0	-

ALDH Adolescent lumbar disc herniation, CI Confidence Interval, ODI Oswestry Disability Index, NRS Numeric Rating Scale, GPE Global Perceived Effect, LDH Lumbar disc herniation.

## Data Availability

The datasets used and/or analyzed during the current study are available from the corresponding author on reasonable request.

## Abbreviations

The following abbreviations are used in this manuscript:

ALDH	Adolescent Lumbar Disc Herniation
LDH	Lumbar Disc Herniation
PROM	Patient-Reported Outcome Measure
ODI	Oswestry Disability Index
NRS	Numeric Rating Scale
GPE	Global Perceived Effect
EPR	Electronic Patient Record
ICD-10	International Classification of Diseases, Tenth Revision
NCSP	NOMESCO Classification of Surgical Procedures
REK	Regional Committee for Medical and Health Research Ethics

## Appendix A

**Table A1:** Individual patient characteristics and postoperative outcome measures.

Age at Time of Surgery (Years)	Gender	Duration of Symptoms (Months)	Follow-Up (Months)	ODI *	NRS Leg Pain **	NRS Back Pain **	GPE ***
17	Female	13	100	4	2	1	2
18	Female	13	104	6	1	3	2
17	Female	18	107	32	7	8	4
16	Male	7	11	2	2	1	1
17	Female	25	105	16	6	2	2
17	Female	7	49	8	1	2	1
18	Female	5	30	6	2	2	1
16	Male	3	121	0	1	1	1
18	Male	5	103	-	4	5	2
18	Female	24	108	12	2	3	2
14	Male	7	42	10	2	3	2
18	Male	4	37	4	1	1	1
18	Male	9	10	24	1	3	2
16	Female	6	75	8	-	-	2
17	Male	16	34	0	2	1	2
16	Male	6	93	10	1	2	3
16	Male	24	22	-	2	7	4

* ODI Oswestry Disability Index, 0–100 (0 = No disability, 100 = Bed-bound); ** NRS Numeric Rating Scale, 1–10 (1 = No pain, 10 = Worst imaginable pain); *** GPE Global Perceived Effect, (1 = “Completely recovered”, 2 = “Much improved”, 3 = “Somewhat improved”, 4 = “Unchanged”, 5 = “Somewhat worse”, 6 = “Much worse”, 7 = “Worse than ever”).

**Table A2:** Patient-reported activity level prior to disc herniation.

		Number	Proportion (%)
Normal activity; no organized sports team		4	23.5%
Exercise regularly 1–3 times per week; no organized sports team		4	23.5%
Exercise regularly 1–3 times per week; affiliated with sports team		2	11.8%
Exercise more than 3 times per week; affiliated with sports team		7	41.2%
	Soccer	1	14.3%
	Boxing	2	28.6%
	Handball	3	42.9%
	Orienteering	1	14.3%

**Table A3:** Comparison of baseline characteristics between Non-Responders and Responders in adolescents treated for lumbar disc herniation at Akershus University Hospital from January 2014 to April 2024.

		Non-Responders N = 10	Responders N = 17
Age, years (95% CI)		16.1 (15.1–17.1)	16.9 (16.3–17.5)
Females, n (%)		8 (80%)	8 (47.1%)
Symptoms, n (%)			
	Leg pain	10 (100%)	15 (88.2%)
	Neurologic deficit	2 (20%)	5 (29.4%)
Disc herniation segment n (%)			
	L5/S1	6 (60%)	11 (64.7%)
	L4/L5	3 (30%)	6 (35.3%)
	L2/3 and L3/4 *	1 (10%)	0
Preoperative symptom duration, months (95% CI)		8.2 (0.4–16.0)	11.3 (7.4–15.2)

* One patient had lumbar disc herniation in both L2/3 and L3/4 segments; CI Confidence Interval.
